# Evidence-Based Practice and Nursing Research

**DOI:** 10.1097/jnr.0000000000000346

**Published:** 2019-07-16

**Authors:** Li-Yin CHIEN

Evidence-based practice is now widely recognized as the key to improving healthcare quality and patient outcomes. Although the purposes of nursing research (conducting research to generate new knowledge) and evidence-based nursing practice (utilizing best evidence as basis of nursing practice) seem quite different, an increasing number of research studies have been conducted with the goal of translating evidence effectively into practice. Clearly, evidence from research (effective innovation) must be accompanied by effective implementation, and an enabling context to achieve significant outcomes.

As mentioned by Professor Rita Pickler, “nursing science needs to encompass all manner of research, from discovery to translation, from bench to bedside, from mechanistic to holistic” (Pickler, 2018). I feel that *The Journal of Nursing Research* must provide an open forum for all kind of research in order to help bridge the gap between research-generated evidence and clinical nursing practice and education.

In this issue, an article by professor Ying-Ju Chang and colleagues at National Cheng Kung University presents an evidence-based practice curriculum for undergraduate nursing students developed using an action research-based model. This “evidence-based practice curriculum” spans all four academic years, integrates coursework and practicums, and sets different learning objectives for students at different grade levels. Also in this issue, Yang et al. apply a revised standard care procedure to increase the ability of critical care nurses to verify the placement of nasogastric tubes. After appraising the evidence, the authors conclude that the aspirate pH test is the most reliable and economical method for verifying nasogastric tube placement at the bedside. They subsequently develop a revised standard care procedure and a checklist for auditing the procedure, conduct education for nurses, and examine the effectiveness of the revised procedure.

I hope that these two studies help us all better appreciate that, in addition to innovation and new breakthrough discoveries, curriculum development and evidence-based quality improvement projects, though may not seem so novel, are also important areas of nursing research. Translating evidence into practice is sound science and merits more research.
